# The Willis F. Westmoreland Memorial

**Published:** 1893-11

**Authors:** B. W. Allen

**Affiliations:** Hurtsboro, Ala.


### THE WILLIS F. WESTMORELAND MEMORIAL.

HURTSBORO, ALA., October 25, 1893.

*To Editor Atlanta Medical and Surgical Journal, Atlanta, Ga.:*

DEAR SIR—In September JOURNAL you published a communication from me with reference to the erection of a monument to the memory of Dr. Willis F. Westmoreland by the alumni of the Atlanta Medical College. Since the publication of said article, I have received sufficient assurance from the *old boys* to warrant me to believe that money requisite for the purpose can be easily raised. It seems to me all that will be necessary is the organization of an efficient committee to take the matter in charge. I would be glad to have any suggestion from you or any of your readers, with reference to the best way to organize or create such committee. I feel very much in earnest about the matter, and would very much like to see the work pushed to completion. There is no nobler work left for the students of Dr. Westmoreland to do than to erect an enduring monument to his memory. I feel sure that my assertion will not, and can not be challenged, that no man ever lived in the South who took more pains and made more personal sacrifices to teach his students than did Dr. Westmoreland. Hoping that you will give this space, and that I will be gratified by soon hearing that an efficient committee has been organized to take charge of the work, I beg to remain, Yours fraternally,

B. W. ALLEN.

[We would be pleased to see a suitable memorial erected to the memory of Dr. Westmoreland by the alumni of the college in which he taught so successfully for so many years. Possibly no teacher ever enjoyed to a greater degree the confidence and esteem of his students, and it would be an expression of their regard, as appropriate as it would be deserved, for them now to pay this final tribute to his memory. It is suggested to us by Dr. H. V. M. Miller, the colleague and friend of Dr. Westmoreland for many years, that a memorial bust would be more appropriate than a monument. An association might be organized for the desired object, and an executive committee appointed, the latter to solicit and receive subscriptions, etc. THE JOURNAL will cheerfully render any assistance in its power.—EDITOR.]

